# The Catheter in Enlarged Prostate

**Published:** 1876-01

**Authors:** 


The Catheter in Enlarged Prostate.—At a meeting of the Tri-States Medical Association, held at the Delaware House, Port Jervis, New York, December 1st, 1875, Dr. W. L. Appley, of Cocheton, New York, read a paper on the treatment of enlarged prostate, advocating the early use of the catheter as the best remedy; giving his experience, and the result of several cases treated by him in this way. Three men over sixty years of age, that had acquired the tolerance of the catheter, and introduced it themselves four or five times daily, and could not void a drop of urine without it, depending upon it entirely to empty the bladder. One had used it two years, one four or five, and one had worn a silver catheter constantly for ten years, removing it once a week long enough to wash it. The man is hale, ruddy-faced, and quite active. The doctor urged it as a duty upon the profession, to be unanimous in insisting upon the early use of the catheter by the unwilling patient; and have him understand the importance of commencing the use of the instrument early, and have him learn to be his own surgeon, and acquire the tolerance of the instrument before he has retention.

The Association voted unanimously to have the paper published.—*Med. and Surg. Reporter.*

